# Short-term effects of positive end-expiratory pressure on breathing pattern: an interventional study in adult intensive care patients

**DOI:** 10.1186/cc3735

**Published:** 2005-06-09

**Authors:** Christoph Haberthür, Josef Guttmann

**Affiliations:** 1 Assistant Professor and head of Surgical Intensive Care Medicine, Department of Anaesthesia, Kantonsspital Luzern, Switzerland; 2 Professor in Biomedical Engineering, Section of Experimental Anaesthesiology, Department of Anaesthesia and Critical Care Medicine, University of Freiburg, Germany

## Abstract

**Introduction:**

Positive end-expiratory pressure (PEEP) is used in mechanically ventilated patients to increase pulmonary volume and improve gas exchange. However, in clinical practice and with respect to adult, ventilator-dependent patients, little is known about the short-term effects of PEEP on breathing patterns.

**Methods:**

In 30 tracheally intubated, spontaneously breathing patients, we sequentially applied PEEP to the trachea at 0, 5 and 10 cmH_2_O, and then again at 5 cmH_2_O for 30 s each, using the automatic tube compensation mode.

**Results:**

Increases in PEEP were strongly associated with drops in minute ventilation (P < 0.0001) and respiratory rate (*P *< 0.0001). For respiratory rate, a 1 cmH_2_O change in PEEP in either direction resulted in a change in rate of 0.4 breaths/min. The effects were exclusively due to changes in expiratory time. Effects began to manifest during the first breath and became fully established in the second breath for each PEEP level. Identical responses were found when PEEP levels were applied for 10 or 60 s. *Post hoc *analysis revealed a similar but stronger response in patients with impaired respiratory system compliance.

**Conclusion:**

In tracheally intubated, spontaneously breathing adult patients, the level of PEEP significantly influences the resting short-term breathing pattern by selectively affecting expiratory time. These findings are best explained by the Hering–Breuer inflation/deflation reflex.

## Introduction

Pulmonary stretch receptors affect the resting respiratory pattern by vagal afferents. Increased stretch receptor activity shortens the duration of inspiration in animals [1], human newborns, and children [2-9]. This reflex is well known as the Hering–Breuer inflation reflex [10]. Increased stretch receptor activity also prolongs expiration, and maintained inflations – if sufficiently large – can produce apnoea for a considerable period of time. In humans the inflation/deflation reflex is substantially weaker than in most animal species [1,8]. In adult humans, the inflation/deflation reflex has been found to become apparent if the inflation volume exceeds a critical threshold of about 1 l [3,9,11]. Recent work revealed that the inflation/deflation reflex is also operative in normal tidal breathing if subject is sleeping [12,13] or under slight sedation [14]. Moreover, Tryfon and coworkers [14] demonstrated that raising the level of continuous positive airway pressure (or positive end-expiratory pressure [PEEP]) significantly prolongs expiration in parallel with a PEEP-related increase in functional residual capacity. However, the findings of those studies [12-14] became apparent only after an inspiratory hold manoeuvre and are therefore beyond the limits of physiological or common clinical conditions.

Our aim in the present study was to investigate whether these effects also become apparent during standard clinical conditions. To this end, we repeatedly applied a pattern of different levels of PEEP in a heterogeneous population of tracheally intubated patients during unsupported spontaneous breathing. Using this approach we were able to demonstrate that increased PEEP was strongly associated with prolongation of the expiratory time (resulting in a fall in both respiratory rate and minute ventilation [V_E_]), whereas tidal volume (V_T_) and inspiratory time were not significantly affected.

## Materials and methods

### Patients

Thirty patients were investigated during weaning from mechanical ventilation. The characteristics of the patients and their underlying diseases are summarized in Table 1. Sedation therapy had been discontinued in 21 (70%) patients 6 ± 7 hours before the start of the study. At entrance into the study, all patients were either awake or easy to arouse, corresponding to a Ramsey sedation score of 2 or 3 [15]. Patients were breathing spontaneously in the pressure support ventilation (PSV) mode with an inspiratory pressure assist of 6–12 cmH_2_O above a PEEP of 5–10 cmH_2_O. For a patient to be enrolled in the study, the fractional inspired oxygen (FiO_2_) required to maintain their arterial oxygen tension and arterial oxygen saturation above 10 kPa (75 mmHg) and 92%, respectively, had to be 50% or less. Furthermore, the patients had to be able to maintain blood gas values within normal ranges, to exhibit no severe coughing or repeated swallowing, or obvious signs of discomfort, and to have stable cardiovascular and respiratory conditions (i.e. absence of systematic changes to ventilatory pattern, FiO_2 _or PEEP requirements, or haemodynamic conditions). Of these patients, 19 (63%) could be successfully extubated within 1–24 hours after termination of the study. The remaining 11 patients (37 %) had to be kept on the ventilator for a mean of 3 ± 2 days for prolonged weaning from mechanical ventilation for various reasons.

### Study design

The study protocol was approved by the ethics committee of our institution, and informed consent was obtained either from the patient (in the case of elective surgery) or from the patient's next of kin. After study inclusion criteria had been fulfilled, patients were connected to a modified study ventilator [16], in which the level of PEEP was set to 5 cmH_2_O. If necessary, FiO_2 _was adjusted to maintain oxygenation within comfortable ranges (i.e. arterial oxygen tension and arterial oxygen sturation above 11 kPa [82.5 mmHg] and 94%, respectively). After 20-min period to stabilize the temperature and humidity of the system and to allow the patient to become accustomed to the ventilator, the ventilatory mode was switched from conventional PSV to the automatic tube compensation mode [16,17]. We used automatic tube compensation mode (i.e. tracheal continuous positive airway pressure) instead of conventional PSV mode in order to eliminate the influence of flow-dependent pressure drop across the endotracheal tube (ETT) [17,18].

The study ventilator was driven by an external control unit, which – in accordance with the study protocol – automatically changes the PEEP level in synchrony with the patient's breathing pattern (i.e. upward steps in PEEP during inspiration and downward steps during expiration). Even though this helps to minimize interference with the patient's breathing pattern, the duration of expiration of the first breath after PEEP adjustment was shortened and prolonged, respectively, by upward steps and downward steps in PEEP. Consequently, these breaths had to be rejected from our analyses.

**Figure 1:**
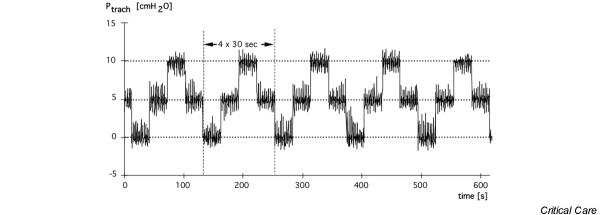
Time course of tracheal pressure during steps in PEEP. The examination was performed while the patient was breathing spontaneously at (tracheal) continuous positive airway pressure by means of the automatic tube compensation mode. Starting from 5 cmH_2_O, the level of PEEP was changed to 0, 5, 10 and (again) 5 cmH_2_O for durations of 30 s each for five consecutive runs. Note that the changes in PEEP level were in synchrony with the patient's breathing pattern (i.e. upward steps during inspiration and downward steps during expiration). PEEP, positive end-expiratory pressure; P_trach_, tracheal pressure.

Instead of airway pressure, the tracheal pressure was the target for PEEP adjustment. To this end, the tracheal pressure was continuously calculated [19] and repeated spot-check measurements between investigations guaranteed for quality of the calculation procedure [20]. The study protocol includes three levels of PEEP that were applied in the following sequence: 0, 5 and 10 cmH_2_O, and the 5 cmH_2_O again. Each level of PEEP was applied for 30 s (Fig. 1). To minimize time-dependent biases, the PEEP sequence was applied five times in a row. Additionally, we applied the study protocol for intervals of 10 s (*n *= 25) and 60 s (*n *= 18).

## Measurements

The flow (V') was measured using a heated Fleisch No. 2 pneumotachograph (Metabo, Epalinges, Switzerland), which was placed at the proximal end of the ETT. Airway pressure was measured between the pneumotachograph and the outer end of the ETT. Spot-check measurement of tracheal pressure were taken by introducing a pressure measuring catheter into the trachea via the lumen of the ETT, as described in detail elsewhere [20]. The pressure transducers used to measure airway and tracheal pressures (32NA-005D; ICsensor, Milpitas, CA, USA) and the differential pressure transducer for measuring V' (CPS 10; Hoffrichter, Schwerin, Germany) were placed a small distance from the patient (20 cm) to achieve a good signal quality and short response time. Expiratory carbon dioxide was measured using a device (CO_2 _Analyzer 930; Siemens-Elema, Solna, Sweden) that was previously calibrated with a carbon dioxide concentration of 8.4%. Measured signals were digitized with 12-bit precision and stored at a rate of 100 Hz in a personal computer for further analyses. The mechanics of the respiratory system (i.e. compliance, resistance and intrinsic PEEP) were calculated from tracings originating from a preceding period of controlled mechanical ventilation [21,22], in which resistance is depicted as pure airway resistance (i.e. without the resistance of the ETT).

### Analysis

Stored data were analyzed on a breath-by-breath basis after having rejected the breaths that were affected by transitions in PEEP level. We determined inspiratory time, expiratory time and respiratory rate by means of the flow signal, in which a combined criterion of flow and expiratory carbon dioxide concentration allowed us to differentiate between inspiration and expiration accurately [23]. V_T _was calculated by numerical integration of V'. V_E _was calculated by multiplying respiratory rate by V_T_. In 10 out of 30 patients, the exhaled carbon dioxide volume per minute was derived from the additive compound of flow and carbon dioxide samples during expiration. For subsequent statistical analysis, data for corresponding PEEP levels and identical time cues were averaged.

In a *post hoc *analysis we investigated the PEEP-related effect on breathing pattern in patients without (control group; *n *= 17) and with impaired lung mechanics (impaired group; *n *= 13). Allocation was roughly based on respiratory system compliance above or below 50 ml/cmH_2_O (Table 2). Because at the time of investigation pure airway resistance (i.e. without ETT resistance) was below 4 cmH_2_O·s/l in each patient, allocation according to airway resistance was not feasible.

Statistical analysis was performed using SYSTAT, version 5.2 (L. Wilkinson, M.A. Hill, E. Vang, Evanston, IL, USA). Differences in all parameters of interest were assessed by an analysis of variance suitable for repeated measures with four repeated within factors (i.e. the PEEP levels) and either no between factor (main analysis) or one between factor (group) in the *post hoc *analysis. Significance was expressed as the arithmetic mean of Greenhouse–Geisser's and Huynh–Feldt's adjusted *P *values. If the overall model showed significant results, then changes between PEEP levels were calculated. For baseline group differences in the *post hoc *analysis, the Kruskal–Wallis test was used to compare continuous variables and the χ^2 ^test was used to compare categorical data. For all tests a two-sided α level of *P *< 0.025 was considered statistically significant. All data are presented as mean ± standard deviation, unless otherwise stated.

## Results

For the investigation with 30 s intervals, a total of 175 ± 88 breaths per patient were eligible for statistical analysis. In each patient the increase in PEEP from 0 to 10 cmH_2_O was associated with a fall in V_E _from on average 11.6 ± 3.0 l/min to 10.0 ± 2.0 l/min (*P *< 0.0001). Whereas V_T _was unaffected by the increase in PEEP (545 ± 184 ml versus 550 ± 163 ml at PEEP 0 and 10 cmH_2_O, respectively; *P *= 0.571), the fall in V_E _was due to a decrease in respiratory rate from 23.6 ± 9.6 breaths/min at zero PEEP to 19.9 ± 7.3 breaths/min at 10 cmH_2_O PEEP (*P *= 0.001). The decrease in respiratory rate was due to a significant increase in expiratory time (from 2111 ± 893 ms at zero PEEP to 2599 ± 1047 ms at 10 cmH_2_O PEEP; *P *< 0.0001), whereas inspiratory time remained unaffected (869 ± 321 ms and 880 ± 312 ms at PEEP 0 and 10 cmH_2_O, respectively; *P *= 0.116). Expiratory carbon dioxide volume significantly decreased with increasing levels of PEEP (*P *< 0.01), whereas the end-expiratory carbon dioxide did not differ significantly.

**Figure 2:**
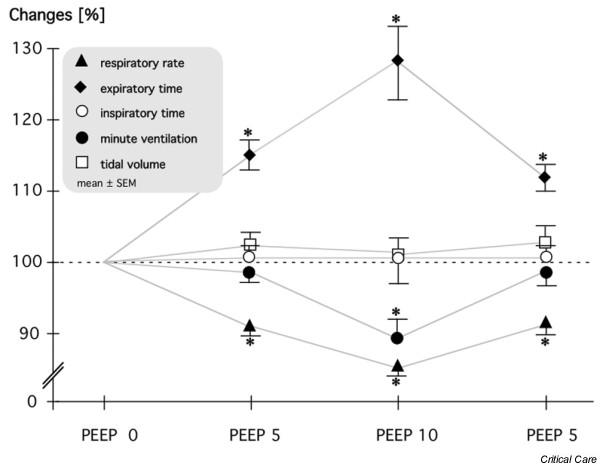
Short-term effects of PEEP on breathing pattern. The increase in PEEP is paralleled by an increase in expiratory time with a concomitant fall in both respiratory rate and minute ventilation. The decrease in PEEP has an opposite effect. Tidal volume and inspiratory time were not significantly affected by changes in PEEP in either direction. Results are outlined as averaged percentage changes from zero PEEP and are expressed as mean ± 1 standard error of the mean (SEM). *P < 0.025, versus zero PEEP. PEEP, positive end-expiratory pressure.

**Figure 3:**
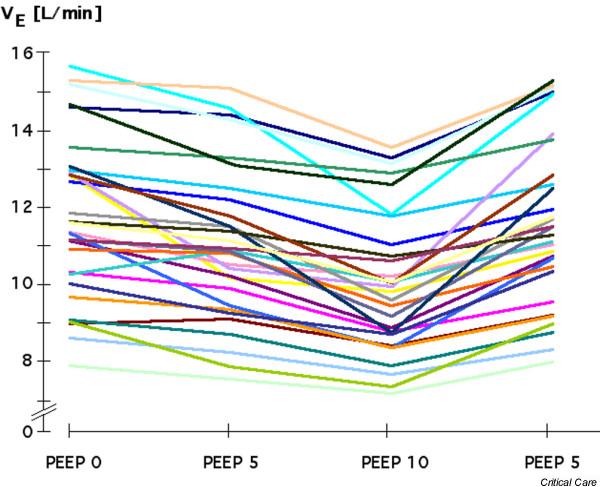
Short-term effects of PEEP on V_E _in the individual patient. Results are from the investigation with PEEP steps of 30 s duration, and each coloured line shows findings for a different patient. Although V_E _is dispersed over a wide range, its behaviour was similar between patients (i.e. V_E _decreased with increasing levels of PEEP, and *vice versa*). PEEP, positive end-expiratory pressure; V_E_, minute ventilation.

**Figure 4:**
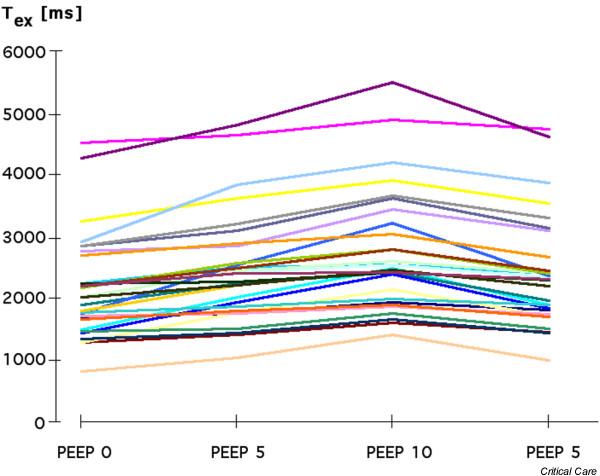
Short-term effects of PEEP on expiratory time in the individual patient. Results are from the investigation with PEEP steps of 30 s duration, and each coloured line shows findings for a different patient. Although T_ex _is dispersed over a wide range, its behaviour was similar between patients (i.e. T_ex _increased with the increasing levels of PEEP, and *vice versa*). PEEP, positive end-expiratory pressure; T_ex_, expiratory time.

**Figure 5:**
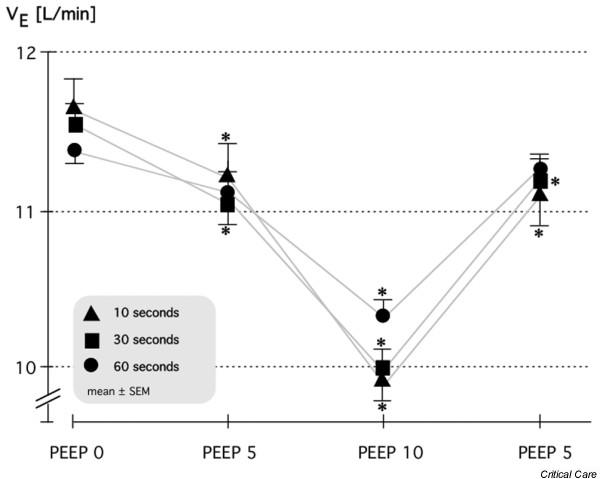
Effects on breathing pattern brought on by steps in PEEP of different duration. Effects are shown on V_E_, which behaved similarly whether the PEEP steps were applied for 10, 30, or 60 s. *P < 0.025, versus zero PEEP. PEEP, positive end-expiratory pressure; V_E_, minute ventilation.

**Figure 6:**
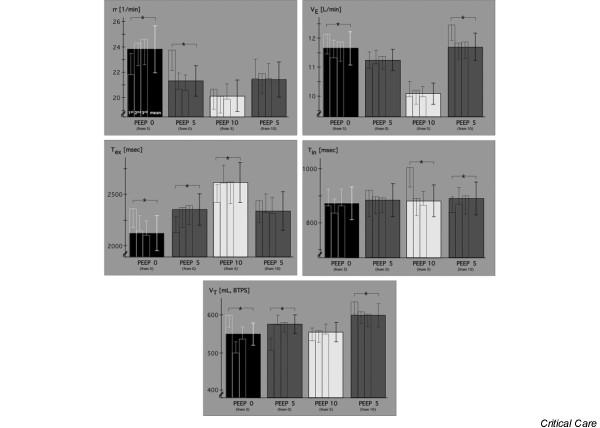
Changes of breathing pattern in the first, second and third breath after steps of PEEP. Results are from the investigation with PEEP steps of 30 s duration. The shaded areas represent averaged values of all breath for the corresponding PEEP level; open bars indicate the first, second, and third breath after changes of PEEP. Values are expressed as means ± 1 standard error of the mean (SEM). Note that changes of breathing pattern were beginning to manifest within the first breath and became fully established in the second breath after both upward and downward steps in PEEP. *P < 0.025, versus mean values within the corresponding PEEP level. BTPS, body temperature pressure, (water damp) saturated; PEEP, positive end-expiratory pressure; rr, respiratory rate; T_ex_, expiratory time; T_in_, inspiratory time; V_E_, minute ventilation.

For the variables of interest, the differences between all levels of PEEP are shown in Table 3; Fig. 2 shows the corresponding percentage changes. Figures 3 and 4 show the PEEP-related effects on V_E _and expiratory time in individual patients. An identical pattern was found when the PEEP pattern was applied for intervals of 10 s or 60 s (Fig. 5). Figure 6 shows changes in breathing pattern in the first, second and third breaths after steps of PEEP in either direction. Changes in breathing pattern had begun to manifest during the first breath and became fully established in the second breath after both upward and downward steps in PEEP.

### *Post hoc *analysis

In patients with decreased respiratory system compliance, respiratory rate and V_E _were significantly higher and inspiratory time, expiratory time and V_T _were significantly smaller as compared with the corresponding parameters in the control group. Irrespective of these group differences, the PEEP-related effect on breathing pattern was the same in both groups (i.e. expiratory time increased, V_E _and respiratory rate decreased, and inspiratory time and V_T _remained unaffected by increases in the level of PEEP, and *vice versa*). Effects were more pronounced in patients with impaired respiratory system compliance than in the control group (*P *< 0.025). In both groups, the changes became fully established at the latest within the second breath after a change in PEEP.

## Discussion

The findings of this clinical study, conducted in a heterogeneous population of adult intensive care patients, indicate that the level of PEEP significantly influences resting short-term breathing patterns by selectively affecting the duration of expiration. Thus, a reduction in PEEP is paralleled by an increase in respiratory rate and subsequently in V_E_, and *vice versa*. According to our findings, the magnitude of the effect is about 0.4 breaths/min per 1 cmH_2_O change in PEEP in either direction, and so it is about 10 times smaller than the effect found in anaesthetized animals [24]. Our findings are in accordance with the results of other studies in adult humans at normal tidal breathing [12-14], in which the findings were attributed to the Hering–Breuer inflation/deflation reflex [14]. In those studies, however, the effect became apparent only with an inspiratory hold manoeuvre. In contrast, we were able to show this effect also at normal tidal breathing under quite common clinical conditions (i.e. without any dedicated respiratory manoeuvre). Furthermore, we were also able to demonstrate that the PEEP-related effect had already begun to manifest during the first breath and became fully established in the second breath after adjustment to PEEP level. Consistent with these findings, we could not find any substantial difference when the study protocol was applied with PEEP durations as short as 10 s and up to 60 s.

The PEEP-related effect upon breathing pattern found in our heterogeneous study population was not only significant but also substantial. However, whether these findings are of clinical importance remains unclear. On the one hand, this is because in our study we focused on short-term rather than on long-term effects. From a theoretical point of view, the short-term responses seen here might simply be offset by a subsequent shift in blood carbon dioxide concentration beyond the time frame of 60 s. Such an hypothesis is strongly supported by the slight but significant decrease in exhaled carbon dioxide volume with increasing levels of PEEP (as a result of the PEEP-related drop in V_E_).

To investigate whether the PEEP-related short-term effect is only transient, we applied our study protocol to 10 of the 28 patients for durations of 180 and 300 s per level of PEEP. Unfortunately, the obtained data were inconsistent for the vast majority of these patients. This was due to the increased rate of artifacts (mainly due to restlessness during awakening) associated with the prolonged duration of investigation. Consequently, because our study was not designed to be a long-term investigation, we are unable to draw any conclusions on whether the observed short-term responses are preserved over time or whether they are offset by slowly adapting reflexes or behavioural responses over a longer period of observation. On the other hand, the PEEP-related short-term effect is sufficiently substantial that it could be detected by attentive clinicians, and so it could be used to adapt ventilatory settings for weaning from mechanical ventilation. Furthermore, short-term effects are of the utmost importance with respect to the automated weaning procedures and closed-loop ventilation strategies that now are increasingly being applied worldwide [25].

The question then arises as to the mechanism by which changes in PEEP can affect the breathing pattern, as found in this study. Based on findings in animals and humans, it is likely that the PEEP-related effect is best explained by the Hering–Breuer inflation/deflation reflex [3,8,10,26-28]. However, other mechanisms are also possible. First, the effect might be due to behavioural responses. At study entrance, all patients were either awake or easy to arouse because sedation had been discontinued 6 ± 7 hours before the start of the study in most patients and continued at a rather slight level in a few patients. Nevertheless, the PEEP-related responses were quite uniform; for example, they became fully established within the first two breaths after PEEP adjustment in virtually all patients. Furthermore, we did not find any difference in the PEEP-related effect between patients off and those who were still on (slight) sedation. In summary, behavioural responses are unlikely, although they cannot be ruled out.

Second, the PEEP-related effect could also be due to responses of the chemical feedback system. Based on both alveolar gas exchange (and thus on functional residual capacity as a function of PEEP) and the circulatory time (being approximately 6–7 s at normal conditions), any change in alveolar gas composition will influence the response of peripheral (mainly oxygen) and central (mainly carbon dioxide) chemoreceptors [3,8,24]. In the absence of lung overdistension the PEEP-related increase in end-expiratory lung volume would ameliorate gas exchange (if there were any effect on gas exchange at all), which then might result in a compensatory downregulation in ventilation. Effects will be stronger in patients with normal than in those with decreased lung compliance. Although such a possibility would fit well the findings of our study, the slight but significant decrease in expired volume of carbon dioxide with increasing levels of PEEP (resulting from the PEEP-related decrease in V_E_) does not support this hypothesis. In addition, if predominantly chemical feedback responses were at work, then different responses would be expected for the different periods of time for which the PEEP levels were applied (i.e. slight responses with durations of 10 s but stronger responses with durations of 60 s). Finally, the occurrence of the effect within the first breath after PEEP adjustment could hardly be accounted for by chemical feedback mechanisms.

A third alternative explanation for the observed PEEP-related effect might relate to persistent inspiratory muscle activity during exhalation [29,30]. The presence of persistent inspiratory muscle activity would prolong the expiratory time either independent of or in addition to the reflex-related response. The effect of persistent inspiratory muscle activity would manifest as a depressing influence on expiratory flow rate. However, because expiratory flow (i.e. peak flow rate and expiratory volume) was unaffected by the level of PEEP (Table 3), any hypothesis based on persistent inspiratory muscle activity as the source of the observed PEEP-related effect must be rejected.

In conclusion, the PEEP-related short-term effect on breathing pattern found in the present is best explained by neuronal reflex mechanisms (i.e. the Hering–Breuer inflation/deflation reflex).

In the *post hoc *analysis we found a similar response to changes of PEEP in patients with normal and those with decreased respiratory system compliance. For the latter, however, the effects were stronger. Consistent with our results, Tryfon and coworkers [14] found a less sensitive reflex in patients with supranormal compliance (i.e. patients with chronic obstructive pulmonary disease) as compared with control individuals (i.e. with relatively lower compliance) or patients with interstitial fibrosis (i.e. with decreased compliance). At first glance these findings are surprising, because with higher respiratory system compliance alterations in PEEP should result in stronger changes in lung volume, and so stronger PEEP-related effects on breathing pattern are anticipated in this setting. The findings of our and Tryfon's study refute this, which might be related to the expiratory muscle recruitment that has been found in awake (but not sleeping) subjects in response to increased end-expiratory lung volume [29,31,32]. Expiratory muscle recruitment due to a PEEP-related increase in end-expiratory lung volume would be expected predominantly at normal rather than at decreased respiratory system compliance. The finding of attenuated PEEP-related effects in patients with normal compliance fit well with this hypothesis. Even if some of our patients were under slight sedation (but easy to awake) during the investigation, the hypothesis might hold true because we did not find any difference in PEEP-related effect between wakeful patients and those under slight sedation.

An alternative hypothesis for our unexpected results centres on the potential occurrence of intrinsic PEEP during the investigation. If this is the case, then intrinsic PEEP would have occurred predominantly in patients with normal rather than in those with decreased respiratory system compliance. Consequently, the increase in external PEEP would have reduced the work of breathing, and thus would have attenuated changes in the PEEP-related effect on breathing pattern predominantly in patients with normal respiratory system compliance (as found in our and Tryfon's study). However, there was no evidence for the occurrence of intrinsic PEEP, at least during the preceding period of controlled mechanical ventilation, in which V_E _was at a similar level as during the observational period of the study. In addition, careful examination of the expiratory flow pattern during the investigation did not suggest expiratory flow limitation as an indirect sign of the occurrence of intrinsic PEEP.

## Conclusion

In tracheally intubated, spontaneously breathing adult patients, the level of PEEP significantly influences the resting short-term breathing pattern by selectively affecting expiratory time. The mechanism is probably based on the Hering–Breuer inflation/deflation reflex. Further studies are needed to address counteracting behavioural and/or slow responses of chemical respiratory control, and therefore to elucidate the clinical importance of our findings.

## Key messages

• 	In spontaneous breathing patients upwards steps in PEEP significantly decrease respiratory rate and minute ventilation whereas downward steps have just opposite effects

• 	The PEEP-related effects are exclusively due to alteration of the expiratory time

• 	Effects become fully established within the first two breaths after PEEP adjustment and went on for minimally one minute

• 	Findings of this study and theoretical considerations strongly suggest a reflex related response by the Hering-Breuer inflation/deflation reflex

## Abbreviations

ETT = endotracheal tube; FiO_2 _= fractional inspired oxygen; PEEP = positive end-expiratory pressure; PSV = pressure support ventilation; V_E _= minute ventilation; V_T _= tidal volume.

## Competing interests

The author(s) declare that they have no competing interests.

## Authors' contributions

CH designed the study, carried out the measurements, performed the statistical analysis, and drafted the manuscript. JG conceived the study, and participated in its design and helped to draft the manuscript. All authors read and approved the final manuscript.

## Figures and Tables

**Table 1:** Patient characteristics

Patient	Sex	Age (years)	Reason for intubation	Underlying disease	Duration MV (days)	BSA (m^2^)	PaO_2_/FiO_2 _(kPa/fraction)	C_rs _(ml/cmH_2_O)	R_aw _(cmH_2_O·s/l)
BP	m	57	Coma	Liver cirrhosis	3	2.1	27	65	1.1
BW	m	68	ACBG	CAD	<1	1.96	36	77	1.8
BP	f	69	ACBG	CAD	2	1.82	29	54	2.5
CA	f	56	Variceal bleeding	Liver cirrhosis	<1	1.87	39	88	1.2
FW	m	58	ACBG	CAD	<1	2.06	30	71	2.1
FA	f	65	Coma	Diabetes mellitus	2	1.81	50	95	2.2
HH	m	70	ACBG	CAD	<1	1.74	45	67	1.8
JH	m	69	ACBG	CAD	<1	2	30	55	2.0
MG	m	65	Valve replacement	Aortic stenosis	<1	1.92	30	64	1.9
PB	m	60	ACBG	CAD	<1	2.2	45	90	1.9
PJ	m	66	ACBG	CAD	<1	1.93	47	73	2.3
SH	f	65	ACBG	CAD	<1	1.86	44	70	2.8
SE	m	78	Coma	Poisoning	6	2.05	30	83	1.9
PW	m	55	Valve replacement	Aortic regurgitation	<1	1.92	43	62	1.7
HM	f	67	ACBG	CAD	<1	1.84	33	90	2.1
HN	m	65	Valve replacement	Aortic stenosis	<1	1.78	56	57	2.3
BR	f	66	ACBG	CAD	<1	1.65	31	90	1.5
CF	m	33	ARI	Pneumonia	5	1.81	13	33	1.9
DM	f	76	ARI	Pneumonia	7	1.9	31	29	2.5
EW	m	53	Resuscitation	CAD, MI	2	1.94	19	47	2.1
FA	m	54	ARI	Pneumonia	12	2.11	25	35	3.0
RS	m	53	ARI/ARDS	Pneumonia	17	2.04	19	21	1.7
MA	m	20	ARI/ARDS	Pneumonia	13	1.88	23	35	1.4
PP	m	31	Resuscitation	CAD, AMI	6	2.15	23	41	2.3
PU	m	51	ARI	Pneumonia	13	1.97	19	48	2.6
SM	m	70	ARI	Pneumonia	4	1.87	14	19	2.1
SE	f	63	ARI/ARDS	Pneumonia	28	1.92	12	25	2.4
OE	f	63	ARI/ARDS	Pneumonia	22	1.92	23	31	1.8
TR	f	68	Coma	CNS^j ^haemorrhage	2	1.89	45	47	2.9
WB	f	53	ARI/ARDS	Pneumonia	10	1.34	32	28	2.7

ACBG, aorto-coronary bypass grafting; AMI, acute myocardial infarction; ARDS, acute respiratory distress syndrome; ARI, acute respiratory insufficiency; BSA, body surface area; CAD, coronary artery disease; CNS, central nervous system; C_rs_, compliance of the respiratory system; FiO_2_, fractional inspired oxygen; MV, mechanical ventilation; PaO_2_, arterial oxygen tension; R_aw_, airway resistance (beyond the resistance of the endotracheal tube).

**Table 2:** Baseline characteristics: control versus impaired respiratory system mechanics

Parameter	Control	Impaired	*P*
Number	17	13	-
Age (years ± SD)	65 ± 17	53 ± 13	<0.015
Sex (m/f)	11/6	8/5	NS
BSA (m^2^)	1.90 ± 0.20	1.91 ± 0.14	NS
PaO_2_/FiO_2 _(kPa/fraction)	38 ± 9	23 ± 9	<0.011
C_rs _(ml/cmH_2_O)	74 ± 14	34 ± 10	<0.001
R_aw _[cmH_2_O·s/l)	0.9 ± 0.4	1.2 ± 0.5	NS
Intrinsic PEEP (cmH_2_O)	0.1± 0.11	0.1 ± 0.2	NS

Shown are the baseline characteristics of patients without (control) and those with impaired respiratory system mechanics (impaired) according to compliance below or above 50 mL/1 cmH_2_O at the time of study entrance (*post hoc *analysis). BSA, body surface area; C_rs_, compliance of the respiratory system; FiO_2_, fractional inspired oxygen; Intrinsic PEEP, intrinsic positive end-expiratory pressure (in addition to external applied PEEP); PaO_2_, arterial oxygen tension; R_aw_, airway resistance (not included the resistance of the endotracheal tube).

**Table 3:** Changes in breathing pattern produced by gradual changes in PEEP

Variable of interest	Changes in PEEP (cmH_2_O)				
	
	from 0 to 5	from 5 to10	from 10 to 5	from 5 to 0	*P*
T_ex _(ms)	229 ± 326	260 ± 400	-271 ± 282	-218 ± 249	<0.001
rr (breaths/min)	-2.6 ± 4.4	-1.1 ± 3.5	1.3 ± 2.4	2.4 ± 4.1	<0.001
V_E _(l/min)	-0.5 ± 1.9	-1.2 ± 1.1	1.1 ± 1.6	0.4 ± 1.3	<0.001
T_in _(ms)	14 ± 73	-3 ± 54	7 ± 57	-18 ± 61	NS
V_T _(ml BTPS)	26 ± 93	-21 ± 40	45 ± 97	-50 ± 41	NS
ee-CO_2 _(%)	0.0 ± 0.2	0.2 ± 0.2	-0.1 ± 0.2	-0.1 ± 0.2	NS
V_ex_-CO_2 _(ml/min)	-27 ± 73	-82 ± 20	67 ± 99	42 ± 106	<0.01
V'_peak.ex _(l/s)	0.08 ± 0.08	0.02 ± 0.09	0.00 ± 0.09	-0.10 ± 0.08	NS
V_T,ex _(ml BTPS)	18 ± 89	-18 ± 42	49 ± 96	-50 ± 40	NS

ee-CO_2_, end-expiratory carbon dioxide concentration; rr, respiratory rate; T_ex_, expiratory time; T_in_, inspiratory time; V_E_, minute ventilation; V_ex_-CO_2_, exhaled carbon dioxide volume (derived from 10 patients); V'_peak.ex_, peak expiratory flow rate; V_T_, tidal volume; V_T,ex_, expired volume per breath.
